# AI/ML in Translation: PhRMA Foundation Trainee Challenge Award

**DOI:** 10.1111/cts.70380

**Published:** 2025-10-23

**Authors:** Amy M. Miller, Emily Ortman, Tonglei Li, John A. Wagner

**Affiliations:** ^1^ PhRMA Foundation Washington DC USA; ^2^ Purdue University West Lafayette Indiana USA; ^3^ Aditum Bio and Tempero Bio Cambridge Massachusetts USA

The PhRMA Foundation and ASCPT's journal Clinical and Translational Science (CTS) partnered on a Challenge Award competition to recognize trainees for outstanding papers addressing artificial intelligence (AI) and machine learning (ML) in clinical and translational science (https://www.ascpt.org/Resources/ASCPT‐News/View/ArticleId/28634/CTS‐Call‐for‐Papers‐PhRMA‐Foundation‐Trainee‐Challenge‐Award). This partnership between ASCPT and the PhRMA Foundation is the most recent in a long series of successful initiatives. CTS and the PhRMA Foundation have a rich history of championing trainees and early career investigators; consequently, this collaboration is a natural evolution in the partnership. In addition, this Challenge Award competition follows our successful AI/ML Special Collection, which demonstrates the wide scope and applicability of AI/ML in translational science (https://ascpt.onlinelibrary.wiley.com/doi/toc/10.1111/(ISSN)1752‐8062.ai‐machine‐learning).

The PhRMA Foundation's expert review committee selected six papers to receive a $5000 Trainee Challenge Award. These trainee first authors are future leaders tackling a timely and challenging topic. Their papers cover a range of topics on the transformative potential of AI/ML, from clinical prediction to a methodological emphasis on model construction, validation, and robustness. Each contribution represents an innovative response to the call and has significant implications for future research. Congratulations to the authors for being selected by a panel of experts in drug development, translational science, and data science following the acceptance of their manuscripts by CTS.

The award‐winning papers are summarized in Table 1.

**TABLE 1:** Brief summary of PhRMA Challenge Award‐winning AI/ML articles.

Title	Award Author	Key highlights	Potential AI application	References
Application of Machine Learning for Predicting Progression‐Free and Overall Survival in Patients with Renal Cell Carcinoma	Caroline W. Grant	Tree‐based ML models performed better than traditional parametric statistical models in predicting progression‐free and overall survival in patients with renal cell carcinoma.	Cancer progression and survival prediction for trial enrichment and risk stratification	[1]
Network Modeling of Biomarker Systems in Liver Steatosis and Fibrosis	Amruta Gajanan Bhat	Ensemble learning based models delineated factors associated with steatosis and fibrosis, including biomarkers, demographics, and hepatic imaging.	AI‐enhanced dose optimization and dosing regimen individualization incorporating imaging measures	[2]
AI‐Driven Variant Annotation of Precision Oncology in Breast Cancer	Kriti Shukla	Integrated multiomic and structural data were used to identify ESR1‐ and EZH2‐associated mutations with functional annotation in breast cancer.	Scalable variant annotation and functional classification	[3]
A Comparison of AI and Population PK Models to Predict Antiepileptic Drug Concentrations Using Therapeutic Drug Monitoring Records	Tae Kyu Chung	AI models, particularly ensemble methods, predicted anti‐epileptic drug concentrations using therapeutic drug monitoring and electronic medical records.	Alternative to conventional population PK models	[4]
Development of a Novel Machine Learning Method for Estimating Lifelong Chronic Disease Progression and Its Applications in Type 2 Diabetes	Yamato Sano	Biomarkers and hazards for mortality and diabetes‐related outcomes in patients with type 2 diabetes suggest that blood pressure and eGFR significantly contribute to estimation of disease progression.	AI‐guided biomarker discovery with potential use in patient selection and endpoint determination	[5]
Out‐of‐Distribution Detection as a Risk‐Control Strategy for Medical Classification Machine Learning Models	Chu Weng	Out‐of‐distribution detection identified subsets of the data with poor prediction performance and higher variance, due in part to underlying demographic factors.	Identifying high‐risk subsets of patient data with poor model performance, enhancing analytic trustworthiness	[6]

Grant et al. [1] applied tree‐based machine learning methods to predict progression‐free and overall survival of renal cell carcinoma patients. They used patient biomarkers as input features and data from more than 1800 patients.

Bhat and Ramanathan [2] predicted liver steatosis and fibrosis based on biomarker features from the liver elastography data of 5494 participants. What is most interesting about their paper is Bayesian network modeling, in which inference relationships among biomarker features were derived.

In their systems biology paper, Shukla et al. [3] demonstrated a novel ML approach to identify genomic variants in breast cancer by integrating multiomics and protein structure prediction. Various types of data and knowledge bases were creatively used and streamlined.

In another study, Chung and Lee [4] compared the predictive performance of ML, including regression, tree‐based ensemble methods, and neural networks, with that of conventional population pharmacokinetic (PK) models. They extracted PK data of several drugs from hundreds of patient records. The study demonstrated comparable results between machine learning and PK models.

Sano et al. [5] reported an interesting deep learning approach to predict disease progression of type 2 diabetes by analyzing clinical data of more than 10,000 patients. The study shed light on specific biomarkers that correlate with diabetic progression over 30 years.

Finally, Weng et al. [6] discussed the implementation of out‐of‐distribution (OOD) detection algorithms in processing biomedical data such as images, transcriptomic data, and time series observations. Finding an “outlier” in a database could facilitate the training of a machine model.

We are incredibly pleased to see these six high‐quality research papers selected for the PhRMA Foundation Trainee Challenge Award in AI/ML. CTS would like to thank the expert panel for evaluating all the manuscripts submitted to the competition. CTS also wants to thank the PhRMA Foundation for its visionary support of an emerging research area that holds so much potential to reshape drug development.

## Conflicts of Interest

J. A. W. is an employee of Aditum Bio and Tempero Bio. The other authors declare no conflicts of interest.
